# Developmental Stage-Dependent Stoichiometric Characteristics of Fine Roots and Their Relationship with Soil Nutrients in *Caragana tibetica* Shrubs in a Desert Steppe Ecosystem

**DOI:** 10.3390/plants15101473

**Published:** 2026-05-12

**Authors:** Yumei Liang, Lu Liu, Min Han, Qi Tian, Limin Yuan, Yong Gao

**Affiliations:** 1 Inner Mongolia Key Laboratory of Desert Ecological System, Inner Mongolia Academy of Forestry Sciences, Hohhot 010010, China; liangym@imau.edu.cn; 2 College of Desert Control Science and Engineering, Inner Mongolia Agricultural University, Hohhot 010010, China; 15034818270@163.com (L.L.); 13314729077@126.com (M.H.); 3 College of Computer Science, Inner Mongolia University, Hohhot 010021, China; cstianqi@imu.edu.cn

**Keywords:** *Caragana tibetica* shrub, C, N, P contents, fine roots and soil, stoichiometric characteristics, developmental stage

## Abstract

Fine roots serve as the primary organs for nutrient and water uptake in plants while also representing a critical pathway for the return of soil nutrients. *Caragana tibetica* is widely recognized as an indicator species marking the transition from grassland to desert. Therefore, investigating the stoichiometric characteristics of its fine roots is of great significance for elucidating plant–soil nutrient cycling in the desert steppe ecosystem. In this study, taking *C. tibetica* shrubs at different developmental stages as the research objects, the methods of in situ sampling in the field and determination of indoor indicators are adopted to study the stoichiometric characteristics of C, N, and P in fine roots and soils at different developmental stages, as well as the relationship between them. The results indicate significant differences in the stoichiometric characteristics of fine roots across different developmental stages (*p* < 0.05). The fine root C:N and N:P ratios are significantly higher in the decline stage, and the C:P ratio is significantly higher in the stable stage. Along with shrub development, soil nutrient accumulation capacity is significantly greater in the growth and stable stages, whereas no significant difference is observed in the soil total phosphorus content. The soil organic carbon is the greatest in the growth stage, and the total nitrogen is greatest in the decline stage. In addition, in the incipient stage, the correlation between total phosphorus in the soil and the stoichiometric characteristics of fine roots is relatively strong. In the stable stage, the correlation between soil organic carbon and total nitrogen and the stoichiometric characteristics of fine roots is relatively weak. In the decline stage, the correlation between total nitrogen in fine roots and soil nutrients in the growth stage is the strongest. The results of this study reveal the differences in stoichiometric characteristics between fine roots and soil across different developmental stages of the *C. tibetica* shrub, highlighting the distinct adaptation strategies of fine roots and soil to environmental changes in each stage.

## 1. Introduction

Fine roots are the most metabolically active and dynamic apical tissues in plants, serving as the primary organs for nutrient and water uptake [1]. They not only constitute an essential reservoir of nutrients and carbon, but also play a critical role in nutrient cycling within ecosystems [2]. As the most active component of the plant’s subterranean architecture, fine roots exhibit a heightened sensitivity to alterations in the soil environment. They exert a significant influence on plant growth, physiological metabolism, and the dynamic equilibrium of soil nutrients, which are pivotal contributors to the flux of nutrients between plants and soil [3].

*C. tibetica*, a dominant shrub widely distributed in the desert grassland areas of the Inner Mongolia Plateau, with its unique root structure and strong vitality, can prevent the continued desertification of local grasslands [4]. The root system of *C. tibetica* is dense, and its stoichiometric characteristics (C:N:P) not only reflect the nutrient utilization strategy of the plant itself, but are also directly related to litter decomposition and soil organic matter accumulation [5,6]. However, at present, our understanding of the variation patterns in *C. tibetica* fine-root chemistry across developmental stages and the coupling mechanism with soil nutrients remains very limited, especially in the fragile desert grassland habitat. Therefore, taking the *C. tibetica* shrub as the research object and studying the nutrient content and stoichiometry of its root system can provide an in-depth understanding of nutrient limitations on plant growth in desert grassland areas.

Carbon (C), nitrogen (N), and phosphorus (P) are fundamental chemical elements that constitute plant life activities [7]. Among them, carbon (C) is the core element for the formation of plant biomass, while nitrogen (N) and phosphorus (P) are the primary limiting elements encountered during plant growth. The carbon-to-nitrogen ratio (C:N) can serve as an indicator for assessing the intensity of carbon and nitrogen metabolism in plants. The carbon-to-phosphorus ratio (C:P) reflects the efficiency of nutrient utilization by plants and influences various physiological and biochemical processes. Meanwhile, the nitrogen-to-phosphorus ratio (N:P) can indicate the elemental limitations encountered during plant growth and development [8]. Therefore, the stoichiometric characteristics of carbon (C), nitrogen (N), and phosphorus (P) in fine roots are pivotal indicators of plant growth conditions and soil nutrient content in ecosystems. They also function as crucial indicators for assessing plant developmental status and for clarifying nutrient interaction during plant growth processes [9,10]. These characteristics not only reflect the plant’s nutrient and water uptake capacity but also indicate its environmental adaptation, further revealing the ecosystem’s nutrient limitation status [11]. At the root level, the C:N ratio of fine roots usually reflects nitrogen use efficiency, while the N:P ratio is considered to indicate the type of nutrient limitation [12]. Although the stoichiometric characteristics of leaves and roots have been reported at the species and community levels, research on the dynamic changes of fine root stoichiometry across different developmental stages within the same shrub species remains scarce [13]. In fact, as plants transition from early growth to maturity and senescence stages, their patterns of photosynthate allocation, root turnover rates, and abilities to compete for and provide feedback on soil nutrients all undergo significant changes [14]. Ignoring the impact of developmental stages may lead to misjudgment of nutrient adaptation strategies in shrubs. Therefore, studying developmental stage-dependent fine root stoichiometry is crucial for accurately understanding the survival strategies of desert shrubs.

The interaction between roots and soil nutrients is not a static equilibrium, but shows stage-specific coupling characteristics as the plant develops [15,16]. In the early growth stages, shrubs typically allocate more carbon to fine roots to rapidly expand their nutrient-capture area. At this time, the N and P concentrations in fine roots may be higher, and the C:N and C:P ratios are lower, reflecting a strong demand for nutrients [17,18]. After entering the maturity stage, root–soil feedback tends to stabilize, and litter released by fine-root turnover may promote the accumulation of soil organic matter and available nutrients, thereby forming a “fertile island” effect [19,20]. However, at the aging stage, root activity declines and the secretion of substances decreases, which may weaken the positive feedback between roots and soil, and even exacerbate nutrient limitations [21]. It is worth noting that desert steppe soils typically exhibit low organic matter content and heterogeneous distribution, making the above-mentioned stage relationships potentially more complex. There have been many studies on the root–soil coupling relationship in herbaceous plants [22,23], but for shrubs of the genus Caragana, how developmental stages regulate the relationship between fine-root stoichiometry and soil nutrient availability remains poorly understood.

This study focuses on the *C. tibetica* shrubs in desert grassland ecosystems, and from the perspective of ecological stoichiometry, it examines the changes in carbon, nitrogen, and phosphorus content, as well as the stoichiometric characteristics of fine roots and soil under the *C. tibetica* shrub at different developmental stages (i.e., the incipient stage, growth stage, stable stage, and decline stage). By revealing the developmental stage-dependent stoichiometric characteristics of fine roots and their synergistic changes with soil nutrients, this study aims to deepen the understanding of nutrient adaptation strategies of shrubs in arid regions and provide a theoretical basis for life stage-based, precise nutrient regulation in the restoration and management of desert grassland vegetation.

In this context, the present study aims to test the hypothesis that the stoichiometric characteristics of fine roots and their coupling with soil nutrients shift significantly across developmental stages. Specifically, we address the following questions: (1) How do C, N, and P concentrations and their ratios in fine roots change from the incipient to the decline stage? (2) How do soil nutrient pools vary with shrub developmental stage? (3) Are the correlations between fine-root stoichiometry and soil nutrients stage-dependent, and which soil factors explain the variation in fine-root stoichiometry at each stage?

## 2. Results

### 2.1. Changes in the Stoichiometry of Fine Roots at Different Developmental Stages

**Figure 1:**
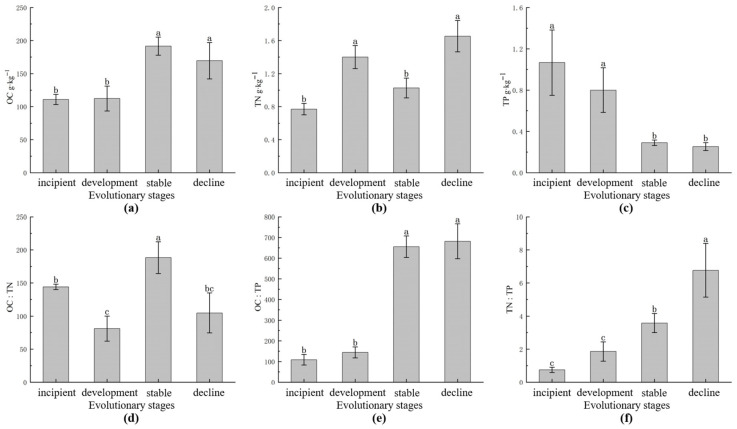
Carbon (C), nitrogen (N), and phosphorus (P) concentrations and stoichiometric ratios in fine roots of *C. tibetica* at different developmental stages. Different lowercase letters indicate significant differences among stages (one-way ANOVA, LSD multiple comparison, *p* < 0.05). (**a**) OC, organic carbon; (**b**) TN, total nitrogen; (**c**) TP, total phosphorus; (**d**) OC:TN, ratio of organic carbon to total nitrogen; (**e**) OC:TP, ratio of organic carbon to total phosphorus; (**f**) TN:TP, ratio of total nitrogen to total phosphorus.

As can be seen in Figure 1, among the four developmental stages, the content of fine root organic carbon (OC) was highest in the stable stage and lowest in the growth stage; the ratios of organic carbon to total phosphorus (OC:TP) in the stable stage and the decline stage were significantly greater than those in the incipient stage and the growth stage, while the TP content in the latter two stages was significantly lower than that in the first two stages (*p* < 0.05). The pairwise differences in fine root organic carbon to total nitrogen (OC:TN) were significant among all stages except between the growth and decline stages (*p* < 0.05), and their magnitudes were in the order of stable stage > incipient stage > decline stage > development stage. OC:TP and TN:TP were significantly greater in both the stable stage and the decline stage (*p* < 0.05), and their magnitudes were in the order of decline stage > stable stage > development stage > incipient stage.

### 2.2. Analysis of Soil Stoichiometric Characteristics at Different Developmental Stages

Analysis of variance revealed that the developmental stage had a significant impact on the stoichiometric characteristics of the soil (Table 1). The differences in soil TP content, soil organic carbon to total phosphorus (SOC:TP), and TN:TP among different developmental stages were not significant (*p* > 0.05), while the differences in the other soil stoichiometric characteristics were significant (*p* < 0.05). The soil organic carbon (SOC) content peaked during the growth and decline stages, reaching 14.6 and 13.75 g·kg^−1^, respectively. The total nitrogen (TN) content was highest in the growth stage, with a value of 0.20 g·kg^−1^. The soil SOC content is lowest in the stable stage, and the TN content is lowest in both the incipient stage and the decline stage. SOC:TN is manifested in the order of decline stage > incipient stage > growth stage > stable stage.

### 2.3. Correlation Analysis of Fine Root-Soil Stoichiometric Characteristics at Different Developmental Stages

In Table 2, it is clear that in the incipient stage, the correlation between the stoichiometric characteristics of C, N, and P in fine roots and the stoichiometric characteristics of soil was higher than that in the other three developmental stages. In the incipient stage, there was a significant negative correlation between the OC content of fine roots and the SOC content of soil (*p* < 0.05). Furthermore, the TN content of fine roots showed an extremely significant positive correlation with soil TN content and the TN:TP ratio (*p* < 0.01), and a significant positive correlation with the SOC:TP ratio (*p* < 0.05). The TP content of fine roots was significantly positively correlated with SOC content and SOC:TP in the soil (*p* < 0.05). The OC:TP of fine roots was significantly positively correlated with soil TN content and TN:TP (*p* < 0.05). In the stable stage, fine root OC:TP was significantly positively correlated with soil TP (*p* < 0.05). The C, N, and P stoichiometric characteristics of fine roots and their analogous characteristics in the soil, respectively, were not significantly correlated during either the growth or decline stage (*p* > 0.05).

### 2.4. Redundancy Analysis (RDA) of Fine Root-Soil Stoichiometric Characteristics Across Different Developmental Stages

To complement the pairwise Pearson correlation analysis presented in Table 2, redundancy analysis (RDA) was applied to simultaneously evaluate the multivariate relationships between fine-root stoichiometric traits and soil properties, and to identify the main edaphic drivers in each developmental stage. Redundancy analysis (RDA) was performed to examine the relationships between the C, N, and P stoichiometric characteristics of plant fine roots and soil physicochemical factors across the four developmental stages. The first two axes of the ecological stoichiometric characteristics in relation to soil physicochemical factors can well reflect the relationship between the environmental stoichiometric characteristics of fine roots and soil physicochemical factors, and this relationship is mainly determined by the first axis.

**Figure 2:**
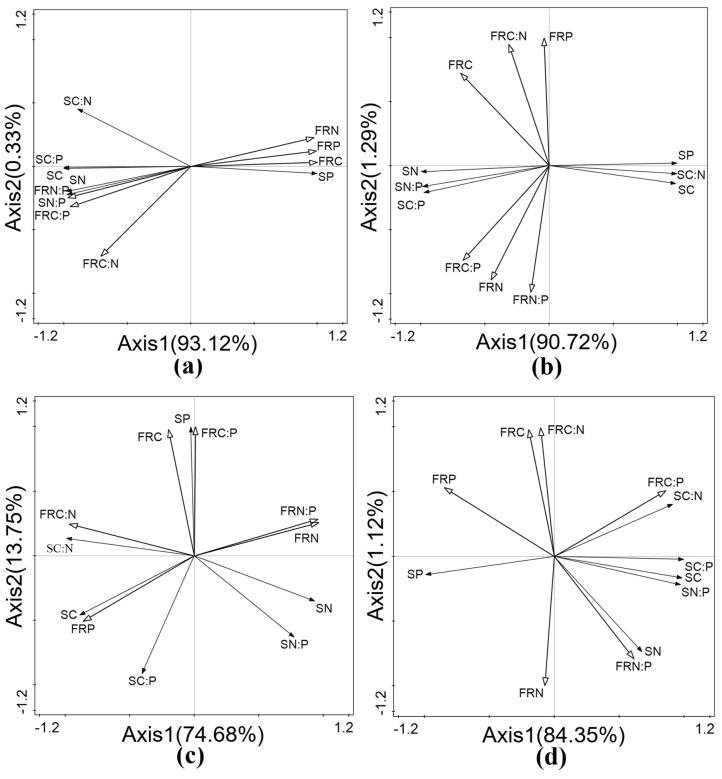
Redundancy analysis (RDA) of fine-root and soil stoichiometric characteristics in (**a**) incipient stage, (**b**) growth stage, (**c**) stable stage, and (**d**) decline stage. Solid arrows represent fine-root stoichiometric variables (FRC, FRN, FRP, FRC:N, FRC:P, FRN:P), and hollow arrows represent soil factors (SC, SN, SP, SC:N, SC:P, SN:P). The length of an arrow indicates the strength of the explanatory power of that variable; the cosine of the angle between arrows reflects their correlation. Solid arrows and hollow arrows are used to indicate the stoichiometric characteristics of the fine roots of *C. tibetica* shrub and soil factors, respectively. The relationship between them is reflected by the length of the connecting lines between the arrows; that is, the longer the connecting line, the greater the correlation between them, and vice versa. Meanwhile, the angle between the connecting lines and the ordination axes also reflects the strength of the correlation: a smaller angle indicates a stronger correlation, whereas a larger angle indicates a weaker one.

Figure 2 displays a two-dimensional ordination diagram illustrating the relationship between the ecological stoichiometric characteristics of fine roots and the physicochemical factors of the soil, where Figure 2a–d present the incipient stage, growth stage, stable stage, and decline stage, respectively.

Figure 2a shows that the arrows representing SN and SP in the incipient stage are the longest, indicating that SN and SP provide a good explanation for the ecological stoichiometric characteristics of fine roots. SP content is positively correlated with the C, N, and P content of fine roots, and negatively correlated with FRC:N, FRC:P, and FRN:P. In contrast, SN shows the opposite relationship, with the weakest correlation observed between SN and fine root C:N (FRC:N).

Figure 2b shows that, compared to other factors, the arrows representing SC, SN, and SP are the longest in the growth stage, indicating that these variables provide a strong explanation for the ecological stoichiometric characteristics of fine roots. SP and SC are not correlated with FRP or FRN:P, and are negatively correlated with other variables of fine roots. In contrast, SN is positively correlated with FRC, FRN, FRP, FRC:N, FRC:P, and FRN:P.

Figure 2c shows that, in the stable stage, the arrow representing SP is the longest, indicating that SP provides a good explanation for the ecological stoichiometric characteristics of fine roots. SP content is negatively correlated with FRP and positively correlated with other ecological stoichiometric characteristics.

Figure 2d shows that, in the decline stage, the arrows representing SP and SC are the longest, indicating that SP and SC provide a good explanation for the ecological stoichiometric characteristics of fine roots. SP is positively correlated with FRN and FRP, with the correlation with FRP being the strongest. SC is positively correlated with FRC:P and FRN:P, and negatively correlated with FRC, FRN, FRP, and FRC:N.

## 3. Discussion

### 3.1. Developmental Changes in Fine-Root Stoichiometry and Their Ecological Implications

Fine-root C:N:P stoichiometry is a key indicator of plant nutrient acquisition strategy and growth limitation. In this study, the low OC:TN and OC:TP observed in the growth stage align with a high nutrient demand during rapid biomass accumulation, whereas the significantly higher OC:TN, OC:TP, and TN:TP in the stable and decline stages reflect a shift toward nutrient conservation and retranslocation. The OC content in roots was highest at the stable stage and then declined, largely because of the shrub’s strong carbon-fixation capacity during the stable phase [24]. The marked increase in root TN during the developmental and decline stages can be attributed to the accumulation of nitrogen-rich reserves [25] and the retranslocation of nitrogen from senescing aboveground tissues [26]. In contrast, root TP remained relatively stable and showed a decreasing trend across stages. The overall stoichiometric ratios followed the order of stable stage > incipient stage > decline stage > growth stage for OC:TN, and decline stage > stable stage > growth stage > incipient stage for OC:TP and TN:TP. These stage-dependent patterns demonstrate that *C. tibetica* fine-root stoichiometry shifts from an acquisitive strategy in early stages to a conservative strategy in later stages [27], and that the shrub becomes co-limited by nitrogen and phosphorus during the decline stage, while such nutrient constraints are less pronounced earlier.

### 3.2. Characteristics of Differences in Soil Element Stoichiometric Ratios at Different Developmental Stages

This study found that the soil’s SOC, TN, and SOC:TN exhibited significant differences (*p* < 0.05) across the four developmental stages. The SOC content in the growth stage and the decline stage was significantly higher than that in the stable stage. TN content was highest in the growth stage, significantly exceeding that in the incipient and decline stages. During the vigorous growth stage, high primary productivity often leads to maximum carbon input [28]. In contrast, the subsequent stable stage may see a balance between input and decomposition, or increased mineralization, leading to lower SOC accumulation [29]. A similar pattern was observed for TN, where the highest content during the growth stage likely reflects strong rhizosphere deposition and rapid nutrient cycling fueled by high root activity. The elevated SOC in the decline stage could be attributed to the massive input of recalcitrant woody debris and reduced microbial activity, a phenomenon observed in late-successional and senescing ecosystems where detrital inputs shift towards more lignin-rich, slow-decaying material, and soil conditions may become less favorable for decomposition [30]. The SOC:TN ratio decreases gradually and then increases as the developmental stage progresses. This can be attributed to the fact that, during shrub growth, the root system continuously secretes various metabolic products—such as amino acids and nucleotides—into the soil, thereby providing nutrients for soil microorganisms [31]. At the same time, the continuous accumulation of litter increases soil organic carbon and nitrogen content, which in turn leads to a close correlation between these two nutrients, as revealed by this study [32]. In the rhizosphere environment, in particular, the influence of root exudates renders nutrient dynamics more complex and intense. These factors interact with one another in the soil ecosystem, influencing its nutrient cycling and plant growth.

### 3.3. Relationships Between Fine Root and Soil Stoichiometric Characteristics Across Different Developmental Stages

Soil, as the primary growth medium for plant roots, significantly influences root stoichiometric characteristics. In turn, roots are highly sensitive to soil quality and environmental changes, adjusting their growth in response to variations in soil moisture and nutrient availability [24]. Analyzing the stoichiometric characteristics of fine roots and soil properties across four developmental stages revealed a significant correlation between them, highlighting the important influence of soil nutrients on fine root growth and development. Different plants have distinct root structures. Even within the same plant, the stoichiometric characteristics of fine roots may vary significantly over time [33]. The growth of the root system is closely related to soil nutrients. The duration of plant growth directly affects the soil’s physical and chemical properties. Meanwhile, variations in soil nutrients among different plant species are also an important factor driving differences in root distribution [34].

These results reveal a significant correlation between the TN content of fine roots and the TN content, TN:TP, and OC:TP in the soil. This is mainly because the chemical composition of fine roots (especially nitrogen content) directly reflects the nutrient status and acquisition strategies of plants, embodying a response relationship between plant internal stoichiometry (such as root C:N:P) and soil nutrients [35,36]. In the stable stage, the OC:TP ratio of fine roots showed a significantly positive correlation with soil TP content. This may stem from the strategy by which plants increase carbon investment to obtain insoluble phosphorus under high-TP conditions [37]. Among the four developmental stages, the fine root stoichiometric characteristics most affected by soil stoichiometric characteristics are the contents of TP and TN, and the one least affected is the content of OC. This is because soil TN and TP, as limiting resources, strongly shape the nitrogen and phosphorus stoichiometry of fine roots through both direct (nutrient supply) and indirect (driving plant carbon allocation strategies) ways [38], while soil OC mainly serves as an environmental matrix. Its content does not directly dictate the carbon concentration of plant tissues [39].

The particularly strong fine root–soil correlations observed in the incipient stage indicate that young *C. tibetica* shrubs are highly dependent on external soil nutrient pools to construct their root systems. As shrubs mature and enter the stable and decline stages, internal nutrient retranslocation and storage become more prominent, gradually decoupling fine-root stoichiometry from ambient soil nutrient concentrations [15,16]. Additionally, the reduced number of significant correlations at later stages may partly reflect increased physiological homeostasis in aging shrubs.

## 4. Materials and Methods

### 4.1. Study Area

The study area is situated in Baiyanhua Gacha, Bilin Temple, Damao Banner, Baotou City, Inner Mongolia (110°10′56″~110°10′57″ E, 42°02′49″~42°02′51″ N). Lying in the northwest part of the Greater Khingan Mountains and the Inner Mongolia Plateau, this region is characterized by a typical desert steppe. The altitude ranges from 1329 m to 1332 m above sea level. The terrain is higher in the south and lower in the north, with a relatively steep southern slope and a gentle northern slope. The soil is predominantly composed of chestnut soil and brown calcic soil, which are slightly alkaline with a pH ranging from 8.0 to 8.5, thereby influencing the solubility and plant availability of phosphorus and micronutrients. The study area is situated in a mid-temperate semi-arid continental climate zone, characterized by dry conditions with scarce and concentrated rainfall. The average annual temperature is 4.2 °C, the average annual frost-free period is 156 days, and the average annual precipitation is 256.2 mm [4,40]. The dominant species include *Caragana tibetica* (shrub), *Stipa capillata* (grass), *Artemisia frigida* (forb), *Stipa breviflora* (grass), and *Leymus chinensis* (grass) [40].

### 4.2. Sample Collection and Processing

The samples were collected in October 2023. Before plot establishment, a comprehensive field survey was conducted to characterize topography, soil types, and vegetation. Based on this survey, four sites representing the incipient, growth, stable, and decline stages of *C. tibetica* shrubs were selected. Each site featured uniform terrain and a vegetation community dominated by *C. tibetica*. Following a comprehensive field survey of topography, soil types, and vegetation, sites representing four developmental stages of *C. tibetica* shrubs were selected based on morphological criteria adapted from previous studies on Caragana shrub demography [4,19]: incipient stage (seedlings or young shrubs with height < 30 cm, no flowering), growth stage (rapidly growing shrubs 30–80 cm tall with obvious new shoots and active flowering), stable stage (shrubs 80–120 cm tall with stable height and crown size, balanced flowering and fruiting), and decline stage (shrubs with a large proportion of dead branches, reduced leaf area, and limited new growth). For each developmental stage, three independent shrub individuals were selected and excavated using the whole-plant method [41]. The entire root system was carefully excavated using the whole-plant excavation method. A trench was dug 1 m from the plant, and soil was removed inward until all fine roots (0–2 mm in diameter) were fully exposed. Because the fine roots of *C. tibetica* are predominantly distributed in the 20–50 cm soil layer, with the highest density at 30–40 cm [4], rhizosphere soil samples were collected from this depth around each shrub using a multi-point mixed sampling approach, passed through a 2 mm sieve to remove impurities, sealed in plastic bags, and transported to the laboratory for determination of carbon, nitrogen, and phosphorus contents.

The collected shrub root systems were gently rinsed several times with tap water to remove surface dirt, and rinsed 2–3 times with distilled water to ensure cleanliness. After the samples were air-dried, the diameter of the root system was accurately measured using a Vernier caliper, and the roots with a diameter of 0–2 mm were classified as fine roots. The dried fine roots were subjected to a killing-green treatment in an oven at 105 °C for 2 h, and then dried at 80 °C to a constant weight. The samples were ground using a grinder, passed through a 100-mesh sieve, collected, and put into sealed bags for the determination of the total carbon, nitrogen, and phosphorus content of the root system. For root samples, total organic carbon (OC) was determined by the potassium dichromate volumetric method with external heating using a programmable electric digestion furnace (ED36, LabTech, Sorisole, Italy) [42]; total nitrogen (TN) was measured by the Kjeldahl method using a Kjeltec 8400 Analyzer (FOSS, Hillerød, Denmark) [43]; total phosphorus (TP) was determined by the molybdenum–antimony colorimetric method using a UV-2600 spectrophotometer (Shimadzu, Kyoto, Japan) [44]. For soil samples, after air-drying and removal of impurities through a 2 mm sieve, the same analytical methods were applied to determine the soil indicators. The content of soil organic carbon was determined by the potassium dichromate volumetric method, the total nitrogen content was determined by the Kjeldahl nitrogen determination method, and the total phosphorus content was determined by the molybdenum–antimony colorimetric method. Stoichiometric ratios (OC:TN, OC:TP, TN:TP) were calculated as mass ratios of the respective elemental concentrations.

### 4.3. Data Processing and Analysis

For each developmental stage, three biological replicates were used for both fine-root and soil variables (n = 3). Before statistical analysis, normality and homogeneity of variances were tested using the Shapiro–Wilk test and Levene’s test, respectively. Data meeting these assumptions were analyzed by one-way ANOVA followed by LSD multiple comparisons. Pearson correlation analysis was used to examine relationships between fine-root and soil stoichiometric characteristics. One-way ANOVA and Pearson correlation were conducted using SPSS 24.0. Results are presented as mean ± standard deviation in tables. Furthermore, we used Canoco 5.0 software to perform redundancy analysis (RDA) for a more intuitive visualization of the complex relationships among the data; the results are shown in the figures.

## 5. Conclusions

This study demonstrates that fine-root stoichiometric characteristics of *C. tibetica* vary significantly across developmental stages, reflecting a shift from nutrient acquisition to nutrient conservation strategies as shrubs age. Soil nutrient pools also exhibit stage-dependent variations, with the highest SOC and TN both occurring in the growth stage. The correlations between fine-root stoichiometry and soil nutrients are strongest in the incipient stage, particularly for TP, and weaken in the stable stage, indicating that root–soil nutrient coupling is not static but dynamically regulated by ontogeny. These findings highlight the need to consider the developmental stage when assessing nutrient cycling and plant–soil feedback in desert steppe ecosystems. For ecological management, targeted nutrient supplementation (e.g., N addition in the growth stage, P regulation in the incipient stage) may enhance the restoration success of *C. tibetica* shrubs in degraded grasslands.

## Figures and Tables

**Table 1:** C, N, and P contents and ecological stoichiometric ratios of the rooted soils of *C. tibetica*.

Developmental Stages	SOC (g·kg^−1^)	TN (g·kg^−1^)	TP (g·kg^−1^)	SOC:TN	SOC:TP	TN:TP
Incipient stage	11.34 ± 3.44 ab	0.11 ± 0.03 b	1.18 ± 0.43 a	100.24 ± 8.04 b	11.13 ± 6.26 a	0.11 ± 0.06 a
Growth stage	14.6 ± 2.18 a	0.20 ± 0.03 a	1.41 ± 0.47 a	76.60 ± 22.57 bc	10.77 ± 2.12 a	0.15 ± 0.07 a
Stable stage	6.70 ± 0.84 b	0.14 ± 0.48 ab	1.21 ± 0.24 a	52.07 ± 19.19 c	5.71 ± 1.54 a	0.12 ± 0.05 a
Decline stage	13.75 ± 3.22 a	0.10 ± 0.14 b	1.22 ± 0.65 a	140.49 ± 22.82 a	15.33 ± 12.54 a	0.10 ± 0.07 a

Different lowercase letters in the same column indicate significant differences among developmental stages (one-way ANOVA, LSD test, *p* < 0.05).

**Table 2:** Correlation of fine root-soil stoichiometric characteristics at different developmental stages.

Developmental Stages	Index	SC	SN	SP	SC:N	SC:P	SN:P
Incipient stage	FRC	−0.999 *	−0.981	0.992	−0.870	−0.993	−0.983
	FRN	−0.990	−0.999 *	0.949	−0.756	−0.997 *	−0.999 *
	FRP	−0.999 *	−0.995	0.976	−0.818	−0.999 *	−0.996
	FRC:N	0.766	0.850	−0.641	0.298	0.808	0.842
	FRC:P	0.973	0.996	−0.917	0.693	0.986	0.994
	FRN:P	0.987	0.999 *	−0.943	0.744	0.996	0.999 *
Growth stage	FRC	−0.804	0.650	−0.716	−0.784	0.483	0.546
	FRN	−0.292	0.500	−0.420	−0.324	0.665	0.608
	FRP	−0.202	−0.023	−0.067	−0.169	−0.225	−0.152
	FRC:N	−0.473	0.263	−0.349	−0.443	0.063	0.136
	FRC:P	−0.536	0.712	−0.646	−0.564	0.839	0.797
	FRN:P	0.027	0.198	−0.109	−0.006	0.392	0.323
Stable stage	FRC	−0.293	−0.530	0.987	0.254	−0.777	−0.769
	FRN	−0.972	0.814	0.220	−0.950	−0.675	0.591
	FRP	0.982	−0.500	−0.605	0.734	0.920	−0.203
	FRC:N	0.733	−0.994	0.286	0.981	0.228	−0.910
	FRC:P	−0.485	−0.342	0.999 *	0.048	−0.891	−0.620
	FRN:P	−0.978	0.799	0.246	−0.941	−0.695	0.569
Decline stage	FRC	−0.393	−0.853	−0.027	0.184	−0.378	−0.416
	FRN	0.133	0.683	0.294	−0.440	0.116	0.157
	FRP	−0.952	−0.953	0.738	−0.623	−0.947	−0.960
	FRC:N	−0.306	−0.801	−0.120	0.275	−0.290	−0.329
	FRC:P	0.739	0.211	−0.953	0.989	0.750	0.722
	FRN:P	0.761	0.996	−0.420	0.272	0.750	0.777

* Indicates significant correlation at the 0.05 level. SC, SN, SP, SC:N, SC:P, and SN:P represent soil OC, soil TN, soil TP, soil OC: soil TN, soil OC: soil TP, and soil TN: soil TP, respectively; FRC, FRN, FRP, FRC:N, FRC:P, and FRN:P represent fine root OC, fine root TN, fine root TP, fine root OC: fine root TN, fine root OC: fine root TP, and fine root TN: fine root TP, respectively. The same abbreviations apply hereafter.

## Data Availability

The raw data supporting the conclusions of this article will be made available by the authors, without undue reservation. The data are not publicly available due to copyright.
